# Factors affecting the provision of health service delivery in schools in Engela district, Ohangwena region, Namibia

**DOI:** 10.4102/hsag.v27i0.2010

**Published:** 2022-09-28

**Authors:** Daniel O. Ashipala, Miina Shapopi

**Affiliations:** 1 Department of General Nursing Science, Faculty of Health Sciences and Veterinary Medicine, University of Namibia, Windhoek, Namibia

**Keywords:** delivery, provision, school health services, thematic analysis, registered nurses, enrolled nurses

## Abstract

**Background:**

While health services have been offered at various schools in Namibia since independence in 1990, coverage has been limited and there has been a notable decline in recent years. This reduction of services is of great concern, with questions being raised regarding what factors are affecting the provision of these services.

**Aim:**

To explore the factors affecting the provision of health services to schools in Engela district, Ohangwena region, Namibia.

**Settings:**

Semistructured interviews were conducted at a public health care facility situated in the northern part of Namibia.

**Methods:**

A qualitative, explorative, descriptive and contextual research design was utilised. Data were collected through semistructured interviews with 15 nurses from a health centre, of whom five were enrolled nurses and 10 were registered nurses. The data were then analysed thematically.

**Results:**

The study revealed three themes: participants’ understanding of school health services; factors affecting the delivery of school health services; and corrective measures for improving the delivery of school health services.

**Conclusion:**

The notable decline in health service provision to various schools within the district suggests that there might be factors affecting the provision of school health services, posing a serious challenge to the active implementation of the School Health Programme.

**Contribution:**

These findings could be used to make adjustments to the provision of school health services and will also serve as an information baseline to elicit suggestions for future research related to school health services.

## Introduction

According to the World Health Organization (WHO) (2016), there is a significant need for health promotion and healthcare services in schools, as over 1.7 million children and adolescents died in 2016 because of preventable illnesses. Globally, 90% of primary school-age children and 80% of lower secondary school-age children are enrolled in school.

The WHO launched the health promoting schools (HPS) initiative to promote health in schools, as they are considered an ideal setting for promoting children’s health (Lee et al. 2019). According to the WHO (2021), an HPS is one that is continually strengthening its capacity to be a healthy setting for living, learning and working.

The adoption of the initiative is regarded as a significant means to deliver on the Vision 2030 Sustainable Development Goals (SDGs) 3 and 4. Sustainable Development Goal 3 strives to ensure healthy lives and promote well-being for all ages, while SDG 4 emphasises ensuring inclusive and equitable quality education and promoting lifelong learning opportunities for all (National Planning Commission [NPC] 2018). The HPS initiative has been effective in improving several aspects of students’ health through six pillars: school policies, physical environment, social environment, health curriculum, involvement with the community and health services (WHO 2021).

Namibia established the School Health Programme (SHP) in 1990 and adopted WHO’s Health Promoting School Initiative (HPSI) in 1998 (Ministry of Health and Social Services [MoHSS] 2015). In 2008, the National School Health Policy (NSHP) was launched by the government to ensure that all school children acquire the knowledge and skills they need to make informed decisions about their health, thereby improving their quality of life.

Namibia has faced several challenges relating to the implementation of SHP, however, regardless of the well-established policy framework. These include a shortage of trained healthcare workers, insufficient health education materials, a lack of equipment for health check-ups and underdeveloped school health information systems (MoHSS 2015). This has led to a notable decline in the provision of services to many northern remote schools (MoHSS 2015). The critical question is what factors are leading to the low number of school visits within the Engela district, as this is posing a serious challenge to the active implementation of the SHP.

A number of studies on school health services provision have been conducted in various countries, but none have been conducted in Namibia. In addition, no studies have explored the experiences of school health nurses in Ohangwena region.

## Purpose

The purpose of this study was to explore the factors affecting the provision of health services to schools in Engela district, Ohangwena region, Namibia.

## Research design

This study employed a qualitative research approach, utilising explorative, descriptive and contextual designs. The qualitative methodology was used because it allows for descriptions and an in-depth understanding of the participants’ experiences (Brink, Van der Walt & Van Rensburg 2018). One of the features of qualitative research is that it is naturalistic and context-based, so it is centred around natural settings where interactions occur (Maree 2018).

### Population and sample

The accessible population in this study was 22 registered and enrolled nurses who were providing school health services to 15 schools in Engela district in Ohangwena region. A purposive nonprobability sampling technique was used to select participants who met the following criteria: (1) be a registered or enrolled nurse providing health services to the seven schools in the catchment area of the health centre; (2) have worked at the health centre for more than 1 year; and (3) be willing to participate and sign an informed consent form. Fifteen (*n* = 15) semistructured telephonic interviews were conducted, which lasted approximately 50 min each. Probing questions were used, and data saturation was reached with the 15th participant.

### Data collection

Data were collected between September and November 2020 by one researcher, who obtained a list of nurses working at the health centre from the registered nurse in charge of the facility. The researcher approached the nurses through a work WhatsApp group, explaining the purpose and nature of the study. Nurses who needed clarification or were interested in participating were requested to contact the researcher directly. Permission for the interviews and audio recording was gained before each interview. The interviews were conducted telephonically because of the COVID-19 pandemic (Lobe, Morgan & Hoffman 2020). Prior to data collection, pilot interviews were conducted with two fourth-year nursing students with the aim of refining the interview guide. These lasted for 45–50 min and no changes were made to the interview guide. Field notes were written immediately after each interview. The interview recordings were transcribed verbatim with the permission of the participants.

The following questions were posed: (1) what is your understanding of school health services? (2) What factors affect the delivery of school health services at your health centre? (3) How could school health services delivery be improved at your facility?

### Data analysis

Raw data from the audio-recordings were transcribed verbatim before they were analysed using Braun et al.’s (2019) six steps of data analysis: (1) familiarisation with the data by rereading interview transcripts, listening to the recordings multiple times and taking notes from the data; (2) generating initial codes in order to describe the content and identify and provide a label for features of the data that were relevant to the research question; (3) searching for themes in the data of all the interview transcripts by reviewing the coded data to identify areas of similarities and overlap; (4) reviewing potential themes by checking them against the organised extracts of the data and if the themes work in relation to the data; (5) defining and naming themes by summarising each theme in a few sentences to produce a coherent and overall account of the data; (6) producing a report by writing a clear, convincing and complex description of the data based on the data analysis. The researcher and an independent coder identified and agreed on the main themes.

### Measures to ensure trustworthiness

The trustworthiness of this qualitative research was addressed based on the four principles framework presented by Lincoln and Guba (1985) in Polit and Beck (2018): credibility, transferability, dependability and confirmability. Credibility was achieved through prolonged engagement, with the researcher spending a month collecting data. Both individual interviews and field notes were used as methods of data collection. In addition, the researcher constantly checked her findings with the participants. Transferability was achieved through dense descriptions, including a comprehensive description of the methods used, including illustrative direct quotes. Dependability and confirmability were achieved by maintaining an audit trail, which is available on enquiry.

### Ethical considerations

Ethical clearance for this study was obtained from the School of Nursing Research and Ethics Committee (SoNREC) by the School of Nursing Research and Ethics Committee of the University of Namibia. The study was allocated ethical clearance number: SoNREC 9/2021. Permission to conduct the study was also granted from the Ministry of Health and Social Services institutional research review board, reference number: 17/3/3MHS. Informed consent was also obtained from each participant prior to data collection, once the aims and objectives of the study were explained. Participation was voluntary and the participants were free to withdraw from the study at any time. Confidentiality and anonymity were ensured by the researcher, who replaced the participants’ names with codes. The data were kept in a computer encrypted with a password only known to the researcher and were only shared with the supervisors.

## Results

The participants included 11 registered and 4 enrolled nurses, all of whom were under the age of 58 and working at the selected healthcare facility. Of these, 10 were female and 5 were male. Each participant had at least 1 year of school health experience.

Three themes were surfaced:

1. participants’ understanding of school health services
2. factors affecting the delivery of school health services
3. corrective measures to improve the delivery of school health services.

### Theme 1: Participants’ understanding of school health services

The participants described school health services as services provided by nurses to school-going children to improve their health and well-being. The subthemes that emerged from this theme were description of school health services, immunisation services, physical examination services, screening services and health education services.

#### Description of school health services

The study participants described school health services as the services offered to school learners by nurses, including physical examinations, immunisations, screenings and health education:

‘[…*N*]urses that go to visit the schools, the schoolchildren, they assess; they do physical examinations, identify problems from the school kids, and if possible, they can treat them; if they can’t treat, they can refer to the nearest clinic or hospital.’ (P6, Male, Registered Nurse)

‘[…*T*]he service that is rendered by nurses to the children at their respective schools … in order to do early detection, treatment and referrals.’ (P10, Female, Registered nurse)

These definitions are in line with WHO’s school health guidelines, which define school health services as those provided by nurses to students enrolled in primary and secondary education, either within the school premises or in health services situated outside the school (WHO 2021). Similarly, Kuponiyi, Amoran and Kuponiyi (2016) reported that effective school health services facilitate early detection and diagnosis with prompt interventions in order to prevent mortality and reduce morbidity. Several studies expressed that school health services provide appropriate opportunities for treatment and solving health, mental, psychological and behavioural problems (Abedi, Abbaszadeh & Motaghi 2016; Bergström et al. 2015).

#### Immunisation services

The participants indicated that they vaccinate school children at 10 years and girls at 15 years and above, as per the immunisation schedule:

‘Yes, we … immunise learners on each visit: diphtheria, tetanus and oral polio vaccines are given to the 10-year-old children of both genders, while tetanus toxoid vaccines are only given to girls from 15 years and above.’ (P2, Female, Enrolled Nurse)

‘Services such as immunisation for 5 years, 10 years and 15 years old learners are given because they do not come to the healthcare facility for immunisations.’ (P12, Female, Registered Nurse)

These findings coincide with a study conducted by Uzoma and Ifeanyi (2019), who highlighted that the immunisation of learners is necessary to prevent illnesses that may lead to morbidity and mortality. The authors were of the opinion that immunisation is a more effective way of using scarce resources, rather than treating diseases after they occur.

#### Physical examination services

The participants highlighted that they are tasked with performing physical examinations on all school-going children to assess if there are any abnormalities requiring immediate treatment or referrals:

‘Physical examination is also performed, such as dental examination for caries, ears inspection for wax impaction, boys for hernias and girls for unperforated vaginal openings, just to mention a few.’ (P12, Female, Registered Nurse)

This is in line with Dibakwane and Peu (2018), who described physical examinations at school as checking for sight, hearing, fine and gross motor, oral and dental issues.

#### Screening services

The participants stated that whenever they perform a screening and find a child with health problems, they decide whether to refer them to a health care facility for nursing management or to a doctor or dentist:

‘Those learners whom we screen and examine and find problems we are unable to manage, we used to write a notice for them on a piece of paper to take to their parents, informing them that they need to take the child to the hospital for treatment.’ (P15, Female, Registered Nurse)

This is as per Bergström et al. (2015) and Uzoma and Ifeanyi (2019), who reported that all learners must be screened to detect abnormalities so that treatment or referral can be initiated before their school performance is negatively affected. Adebayo, Makinde and Omode (2018) argued that school health referrals help to fast-track medical interventions for learners with critical conditions, reduce absenteeism and prevent the spread of infectious diseases.

#### Health education services

The participants indicated that they also provide appropriate and comprehensive health information to the learners:

‘We provide health talks on topics such as teenage pregnancy, sexual transmitted infections, including HIV; those are the topics covered mostly during the school health visit.’ (P10, Female, Registered Nurse)

The provision of health education as described by the findings of this study is in line with the WHO’s school health guidelines, which state that school-going children can benefit from health education related to hygiene and reproductive and sexual health as they transition from childhood to adulthood. Similarly, Uzoma and Ifeanyi (2019) emphasised that health education gives learners the opportunity to understand the implications of not caring for their health.

### Theme 2: Factors affecting the delivery of school health services

This theme relates to the delivery of school health services by the health centre, with the following subthemes: shortage of nurses, lack of school health transport, lack of school health supplies, lack of privacy and school management issues.

#### Shortage of nurses

According to the participants, a persistent shortage of nurses results in them being overburdened with other responsibilities of patient care; as a result, they neglect school health services:

‘The shortage of nurses is really a concern; this thing of multiple services provided by one nurse can be exhausting, although sometimes we are given a cleaner to help us, and it is useless because a cleaner cannot perform the activities that is supposed to be done by a nurse. If the learners are a lot, one nurse cannot examine them all but can just examine few and go back to the facility.’ (P14, Male, Registered Nurse)

This is in accordance with Mohlabi, Van Aswegen and Mokoena (2010), who argued that whenever a shortage of personnel is experienced in primary healthcare facilities in South Africa, managers withdraw school health nurses to replace the missing staff members.

#### Lack of school health transport

The participants observed that there is a lack of school health transport at their health facility:

‘[…*W*]e are just having one transport, and that transport will be taking a patient – let me say we have an emergency, and the available transport will take that patient to the hospital, and the school health will be cancelled for that day.’ (P7, Male, Enrolled Nurse)

The findings of this study correspond with those of a study conducted in South Africa by Shung-King, Orgill and Slemming (2013), who indicated that a lack of fixed school health transport significantly affects the ability of school health teams to reach schools. The findings of this study revealed that school health services are cancelled when there is no transport, unlike in South Africa where nurses use public transport at their own cost to get to and from the schools.

#### Lack of school health supplies

The participants described a lack of essential supplies as a result of the integration of primary healthcare (PHC) services at their facility:

‘At some point we used to experience a stock-out of gloves used for physical examinations, and the vaccines are sometimes not available, and we find ourselves with many learners who missed their schedule, resulting in the increased number of learners to be immunised during the following visit.’ (P2, Female, Enrolled Nurse)

A similar study in South Africa also discovered that a lack of resources and the poor-quality equipment used by school health nurses compromises the quality of care offered to school children (Dibakwane & Peu 2018). Another study by Haider, Ahmad and Ahmed (2018) found that a lack of school health infrastructure, such as properly ventilated classrooms and insufficient medicines, vaccines and sanitation facilities, serves as a key barrier to the provision of school healthcare services. One study described a lack of planning at the national level as a reason for inadequate school health supplies (Saito et al. 2014).

#### Lack of consultation rooms

Furthermore, the participants stated that there is a lack of consultation rooms, which compromises the privacy of the learners when performing physical examinations:

‘The place itself is not really fit to do physical examination, because sometimes there is no curtains to cover the window; there is no bed where you will do physical examination.’ (P9, Male, Registered Nurse)

These findings relate to those reported by Dibakwane and Peu (2018), who indicated that school health nurses need more space when offering services such as physical examinations, screening and health education. The findings of the study also correlate with those of Kuponiyi et al. (2016), who argued that the lack of space for healthcare in many state schools limits the comprehensive delivery of services to the targeted recipients.

#### School management issues

A number of participants claimed that the education sector needs to be engaged on issues concerning school health in an effort to bring about change:

‘OK, sometimes there is a lack of communication with the school management. As a result of increased workload, some school principals may forget to check the school health programme on the noticeboard in order to inform the learners in advance that the service will be available at school on that particular day.’ (P4, Male, Registered Nurse)

This indicates that there is a lack of support and collaboration from school management, yet school health teams are not denied access to render services at the schools. This is in contrast to a study conducted by Mohlabi et al. (2010) in South Africa, which discovered that some school principals refuse to allow nurses entry to their school premises. They argue that school health services belong to the Department of Health and not to the Department of Education; therefore, nurses should not enter schools.

### Theme 3: Corrective measures to improve the delivery of school health services

This theme describes corrective measures for improving the delivery of school health services.

#### Ministry of Health should recruit school health nurses

The participants suggested that the MoHSS should recruit enough nurses to provide school health services:

‘I would recommend that the Ministry of Health … employ school health nurses, and our supervisors should make use of nurses responsible for school health.’ (P9, Male, Registered Nurse)

The recommendations from a study by Dibakwane and Peu (2018) concurred with these findings. They suggested that the government should employ new nurses or those who have retired as additional human resources. A similar recommendation from Mohlabi et al. (2010) was that a dedicated school health team should be allocated within the PHC system for the rendering of school health services.

#### Ministry of Health to procure school health transport

The participants also argued that the MoHSS should arrange for fixed school health transportation:

‘[…*W*]e want the MoHSS to provide specific transport for the school programme; even we have any emergency, it will not affect the school health programme.’ (P12, Female, Registered Nurse)

This is as per Dibakwane and Peu (2018), who suggested that the government sectors involved in the SHP are mandated to cooperate with and innovate national policies. Action plans are needed to launch and sustain the re-engineering of primary health through collaboration between and across the involved sectors and departments.

#### Ministry of Health to provide school health supplies

The participants recommended that ministry should try by all means to have enough supplies of school health materials, including vaccines:

‘I am recommending the Ministry of Health to have resources specifically for the school health services.’ (P6, Male, Registered Nurse)

This recommendation is in line with the WHO’s guidelines (2021), which found that resource implications need to be carefully identified, examined and met. Similarly, Banfield, McGorm and Sargent (2015) argued that the provision of resources should be improved so that there is improved space for services and a consistent supply of health promotion tools and equipment.

#### Ministry of Health to establish consultation rooms for school health services

Moreover, the participants suggested that the Ministry of Health should facilitate the establishment of a school health room in each school:

‘I am thinking that the Ministry of Education should help us with the site for physical examination, because we want to perform comprehensive physical examination but [*have*] no private room to do the examination; we thus only used to perform few examinations, like oral ones, ear examination but no examination of the genitals, because there is no privacy.’ (P11, Female, Enrolled Nurse)

This recommendation echoes that of Factory (2018), who suggested that there is a need for system strengthening and financial support for the construction of hygiene, sanitation and school health infrastructure. Similarly, Keothaile (2016) recommended that private spaces should be created at all schools, which would ensure and improve the provision of health services by health officials.

#### School management’s involvement

The participants proposed deeper involvement of school management and educators on issues related to school health:

‘Learners should be well informed by their teachers when there is school health, so they come with their health passport.’ (P10, Female, Registered Nurse)

‘School principals and life skills teachers need training regarding school health services in order for them to help the nurses and also to know their roles and expectations.’ (P15, Female, Enrolled Nurse)

The recommendation of the study regarding the involvement of school management in SHP activities is similar to that of Keothaile (2016), who suggested that school management should be involved so that the sustainability of a SHP is not compromised. Similarly, Chidiebere et al. (2016) observed that it is crucial to strengthen the training of teachers on school health-related aspects so that teachers know their school health roles.

### Strengths, limitations and areas for further research

The strengths of this study include that the participants were interviewed during times that were convenient to them, and they were allowed to communicate in the language of their choice. As telephonic interviews were used, nuances of body language that may have been otherwise apparent could have been missed. Video calls would have improved the trustworthiness of the study. Even though the study results cannot be generalised, certain areas were identified by the researcher as requiring further research, including nurses’ experiences regarding the utilisation, monitoring and evaluation of school health services at health facilities and schools, as well as the implementation of school health policy guidelines at health facilities and schools.

### Recommendations

Based on the findings of the study, the following recommendations are made:

- The MoHSS should recruit more nurses meant for the smooth running of school health services delivery. To achieve this, replacing those who resign or retire timeously is key so as to avoid a shortage of nurses.
- A sufficient number of nurses must be hired to ensure that all types of health care services are provided, and all learners are covered.
- The MoHSS should procure fixed transport that is specially assigned for school health services delivery and a driver.
- The MoHSS should provide all the essential supplies required for the functioning of the school health programme.
- Collaboration between the MoHSS and others, such as the MoE, the private sector and nongovernmental organisations, needs to be strengthened in order to establish a united effort for promoting the delivery and utilisation of school health services.
- Written instructions based on the SHP guidelines should be made compulsory in all schools, and every school should develop its own health-related policies to enhance the quality and continuity of school health services.
- The SHP is limited by financial and human resources; therefore, the MoHSS should mobilise resources from other ministries, nongovernmental organisations (The United Nations Educational, Scientific and Cultural Organization, United Nations Children’s Fund, the WHO, etc.) and the private sector. Effective collaboration with these organisations will assist the government to implement school-based health programmes in an efficient and practical manner, in order to achieve the health and well-being of school-going children.
- Community members should be used to establish school health committees, which can be utilised to increase school health awareness and identify health problems that are affecting the learners in their communities.

## Conclusion

In the final analysis, school health services play a crucial role in the management of health conditions facing school-going children. Therefore, the implementation of the programme should be an ongoing process to safeguard the health status of learners. The current lack of school health supplies results in poor school health provision; thus, the Ministry of Health and its relevant health divisions and subdivisions – largely the PHC division – need to address the identified shortcomings to ensure that the future delivery of school health services is successful. It is of paramount importance that all stakeholders in the school health domain work collaboratively towards the common goal of promoting the good health of learners. The findings from this study contribute new knowledge regarding the provision of school health services and can be used to adjust their provision.
